# Urological Causes Mimicking Acute Abdomen: Diagnostic Challenges in General Surgical Emergency Practice

**DOI:** 10.7759/cureus.112695

**Published:** 2026-07-15

**Authors:** Naveen Ch, Prabhat Kumar N, Trisha C, Ramachakra Kushal Thota, Muralidhar Chinnapaka

**Affiliations:** 1 Department of General Surgery, Dr. Pinnamaneni Siddhartha Institute of Medical Sciences & Research Foundation, Vijayawada, IND; 2 Department of Internal Medicine, NIMRA Institute of Medical Sciences, Vijayawada, IND; 3 Department of Internal Medicine, Chalmeda AnandRao Institute of Medical Sciences, Karimnagar, IND; 4 Department of Internal Medicine, Gandhi Medical College and Hospital, Secunderabad, IND; 5 Department of Pharmacology, Government Medical College and Hospital Maheshwaram, Hyderabad, IND

**Keywords:** acute abdomen, acute pyelonephritis, diagnostic challenge, emergency surgery, obstructive uropathy, ureteric colic, urological emergency

## Abstract

Background

Urological disorders are important differential diagnoses in patients presenting with an acute abdomen to a general surgical emergency unit. Conditions such as ureteric colic, obstructive uropathy, acute pyelonephritis, urinary retention, renal trauma, and testicular torsion may simulate appendicitis, intestinal obstruction, biliary colic, or perforation peritonitis. This clinical overlap may delay correct diagnosis, particularly when urinary symptoms are subtle or absent. This study aimed to assess the pattern of urological conditions mimicking acute abdomen and the diagnostic difficulties encountered during initial surgical evaluation.

Materials and methods

This hospital-based observational study included patients who presented with acute abdominal pain to the general surgery emergency department and were later confirmed to have a urological cause. Data regarding age, sex, presenting symptoms, initial clinical impression, urine findings, renal function tests, ultrasonography, computed tomography findings, final diagnosis, treatment given, and outcome were recorded and analyzed.

Results

A total of 80 patients were included. Most patients were male subjects (n=56; 70%), and the commonest age group was 31 to 50 years (n=34; 42.5%). Acute abdominal pain was present in all patients, followed by vomiting 42.5%, fever 32.5%, dysuria 28.8%, flank pain 45.0%, and hematuria 21.3%. The most frequent urological cause was ureteric calculus with ureteric colic (n=36; 45.0%), followed by acute pyelonephritis (n=14; 17.5%), obstructive uropathy (n=11; 13.8%), acute urinary retention (n=8; 10.0%), renal or ureteric trauma (n=6; 7.5%), and testicular torsion presenting as lower abdominal pain (n=5; 6.2%). At first assessment, 31 (38.8%) patients were suspected to have a primary general surgical condition. Ultrasonography supported the diagnosis in 58 (72.5%), while computed tomography was required in 29 (36.3%) patients. Conservative treatment was sufficient in 44 (55%) patients and 36 (45%) required urological intervention.

Conclusion

Urological emergencies can closely resemble acute surgical abdomen. Careful urinary history, focused examination, urine analysis, renal function assessment, ultrasonography, and selective computed tomography help in early diagnosis and prevent unnecessary surgical intervention.

## Introduction

Acute abdomen is a common presentation in general surgical emergency practice and usually raises suspicion of conditions such as acute appendicitis, intestinal obstruction, perforation peritonitis, biliary disease, or pancreatitis. However, several urological disorders may present with similar clinical features and can be mistaken for primary surgical abdominal emergencies. This diagnostic overlap is clinically important because delay in identifying a urological cause may lead to inappropriate treatment, delayed referral, unnecessary investigations, or avoidable surgical exploration [1,2].

Pain arising from the kidney, ureter, bladder, prostate, or testis may be perceived as abdominal pain because of shared visceral innervation and referred pain pathways. Ureteric colic, acute pyelonephritis, obstructive uropathy, acute urinary retention, renal trauma, and testicular torsion are some of the important urological conditions that may mimic acute abdomen. These patients may present with abdominal pain, vomiting, fever, guarding, abdominal distension, or lower abdominal tenderness, making the initial clinical diagnosis difficult [3,4].

Ureteric calculus is one of the most frequent urological causes of acute abdominal pain. Depending on the site of obstruction, it may resemble biliary colic, appendicitis, intestinal colic, or pelvic inflammatory pathology. Although flank pain, hematuria, dysuria, and radiation of pain to the groin are useful clinical clues, these symptoms may be absent or not clearly expressed by the patient at presentation [5-7]. In many emergency settings, such patients are first evaluated by general surgeons because abdominal pain is the dominant complaint.

Acute pyelonephritis and obstructed infected systems are particularly important because they may progress rapidly if not recognised early. Fever, abdominal tenderness, leukocytosis, pyuria, hydronephrosis, raised creatinine, or sepsis in the presence of urinary obstruction should alert the clinician to a possible urological emergency. Early imaging and timely decompression by ureteric stenting or percutaneous nephrostomy may be lifesaving in such cases [8-10].

Acute urinary retention may also present as severe lower abdominal pain and distension, especially in elderly men with prostatic enlargement or patients with urethral stricture, neurogenic bladder, clot retention, or drug-induced retention. Simple bedside assessment, suprapubic examination, and catheterisation when indicated can quickly confirm the diagnosis and relieve symptoms [11]. Similarly, testicular torsion may initially present as lower abdominal or groin pain in young males. Failure to perform a genital examination may delay diagnosis and compromise testicular salvage [12,13].

Laboratory evaluation and imaging are essential in differentiating urological mimics from a true surgical abdomen. Urine routine microscopy, renal function tests, complete blood count, and inflammatory markers provide useful diagnostic information. Ultrasonography is often the first-line imaging tool, as it can detect hydronephrosis, renal calculi, bladder distension, urinary retention, and pyonephrosis. However, computed tomography (CT) is more useful in atypical cases, suspected ureteric stones, trauma, or when the diagnosis remains uncertain after initial evaluation [14-18].

The present study was undertaken to assess the spectrum of urological conditions presenting as acute abdomen in a general surgical emergency setting. It also aims to identify common diagnostic challenges and highlight the importance of focused urinary history, clinical examination, urine analysis, renal function assessment, ultrasonography, and selective use of CT in improving early diagnosis and avoiding unnecessary surgical intervention.

## Materials and methods

Study design

The present study was designed as a hospital-based observational study to evaluate urological conditions that presented with clinical features suggestive of acute abdomen in a general surgical emergency setting. The study was mainly descriptive in nature and focused on identifying the pattern of urological diseases that initially appeared similar to common general surgical emergencies. Patients who came with acute abdominal pain and were later confirmed to have a urological cause were carefully assessed to understand the diagnostic difficulties faced during initial evaluation.

Place of study

The study was conducted at Chalmeda AnandRao Institute of Medical Sciences, Karimnagar, Telangana, India. The cases were recruited from patients attending the general surgery emergency department. Since abdominal pain is commonly evaluated first by the general surgery team, this setting provided a suitable clinical background to study urological conditions that may resemble acute surgical abdomen. Patients who required further urological opinion or intervention were evaluated in coordination with the urology department.

Study duration

The study was carried out from 01 January 2025 to 31 December 2025. All eligible patients who presented during this study period and fulfilled the inclusion criteria were considered for enrolment. The duration was selected to obtain an adequate number of cases with different urological presentations encountered in routine emergency practice.

Study population

The study population included patients who presented to the general surgery emergency unit with acute abdominal pain or symptoms suggestive of acute abdomen and were subsequently diagnosed to have a urological cause. Patients presented with symptoms such as abdominal pain, flank pain, lower abdominal discomfort, vomiting, fever, abdominal distension, dysuria, hematuria, urinary retention, or features of sepsis. At our institution, adolescents presenting with acute abdominal pain are initially evaluated in a mixed emergency setting and are triaged to the general surgery emergency team when features of acute abdomen are suspected. Therefore, adolescent patients fulfilling the study criteria were included in the analysis. In pediatric and adolescent patients, ultrasonography was preferred as the initial imaging modality. CT was used selectively and only when ultrasound was inconclusive, clinical suspicion remained high, or complications were suspected. This approach was followed to limit unnecessary radiation exposure while maintaining diagnostic accuracy. Only those patients whose final diagnosis was confirmed as urological in origin were included for analysis.

Sample size

The sample size was estimated using the formula n = Z²pq/d². A 95% confidence level was considered, with Z=1.96. The expected proportion of urological causes among patients presenting with acute abdomen was taken as 10%, with q=1−p and an absolute precision of 7%. Based on this calculation, the minimum sample size required was about 70 patients. To allow for incomplete records and to strengthen the study observations, 80 consecutive eligible patients were finally included during the study period. 

Inclusion criteria

Patients of any age group who presented to the general surgery emergency department with acute abdominal pain or clinical features suggestive of acute abdomen were considered for inclusion. Patients who were initially suspected to have a general surgical condition but were later found to have a urological cause were included. Diagnosis was confirmed through clinical examination, urine analysis, renal function tests, imaging findings, urological consultation, operative findings where applicable, and response to treatment. Patients or legally acceptable attendants who provided consent for participation were included in the study.

Exclusion criteria

Patients with confirmed non-urological causes of acute abdomen, such as acute appendicitis, intestinal obstruction, perforation peritonitis, acute pancreatitis, acute cholecystitis, or other gastrointestinal surgical conditions, were excluded. Patients with chronic abdominal pain without an acute emergency presentation were not included. Cases with previously diagnosed urological disease already under active treatment before the emergency visit were also excluded to avoid duplication of known cases. Patients with incomplete clinical records, inadequate investigation details, or lack of consent were excluded from the final analysis.

Method of data collection

Data were collected using a structured case record proforma prepared for the study. After obtaining consent, demographic details such as age, sex, residence, and relevant clinical history were recorded. Presenting complaints including site and duration of abdominal pain, radiation of pain, vomiting, fever, dysuria, hematuria, frequency of micturition, reduced urine output, urinary retention, history of trauma, and previous history of stone disease were documented.

Each patient underwent a detailed clinical assessment at presentation. General examination included pulse rate, blood pressure, temperature, hydration status, pallor, and features of systemic infection or shock. Abdominal examination included assessment of tenderness, guarding, rigidity, distension, renal angle tenderness, suprapubic fullness, and palpable bladder. In male patients, particularly those with lower abdominal pain, groin pain, or unexplained abdominal symptoms, external genital examination was performed wherever clinically indicated to rule out testicular torsion or other scrotal pathology.

Clinical assessment

At the time of admission or emergency evaluation, the initial clinical impression made by the general surgery team was recorded. This included suspected diagnoses such as appendicitis, intestinal colic, obstruction, perforation, biliary colic, pancreatitis, nonspecific acute abdomen, or suspected urinary tract pathology. Patients in whom urological involvement was suspected were evaluated further with relevant laboratory tests and imaging.

Urological consultation was obtained whenever clinical findings, urine examination, renal function tests, or imaging suggested urinary tract involvement. The final diagnosis was made after correlating clinical features with laboratory investigations, imaging findings, specialist opinion, intraoperative findings where applicable, and clinical response to treatment. This helped in identifying cases where urological diseases closely resembled common surgical emergencies.

Laboratory investigations

Routine laboratory investigations were performed depending on the clinical condition of the patient. Complete blood count was done to assess leukocytosis, anemia, or evidence of infection. Blood urea and serum creatinine were measured to evaluate renal function and to identify renal impairment due to obstruction, infection, dehydration, or trauma. Serum electrolytes were assessed in selected cases, particularly in patients with vomiting, sepsis, renal dysfunction, or urinary obstruction.

Urine routine examination and microscopy were performed in most patients and were useful in detecting hematuria, pyuria, crystalluria, pus cells, red blood cells, and features suggestive of urinary tract infection. Urine culture and sensitivity were advised when infection was suspected, especially in patients with fever, pyuria, sepsis, obstructive uropathy, or recurrent urinary complaints. Blood culture, blood sugar, coagulation profile, and other biochemical investigations were performed when clinically required.

Imaging evaluation

Ultrasonography of the abdomen and pelvis was used as the first-line imaging investigation in most patients. It helped in identifying hydronephrosis, renal calculi, ureteric dilatation, bladder distension, urinary retention, pyonephrosis, renal abscess, and other urinary tract abnormalities. Ultrasound was also useful because it is safe, easily available, inexpensive, and can be performed quickly in emergency settings.

CT was advised in selected cases where ultrasonography was inconclusive, where ureteric calculus was strongly suspected, or where the clinical presentation was atypical. Non-contrast CT was preferred in suspected urinary stone disease because of its ability to detect ureteric calculi and the level of obstruction. Contrast-enhanced CT was performed only when clinically required and when renal function permitted, especially in cases of trauma, suspected complications, or when differentiation from general surgical causes remained difficult. X-ray abdomen, chest X-ray, scrotal Doppler ultrasound, or other imaging modalities were used based on individual clinical indications.

Final diagnosis

The final diagnosis was established by integrating clinical findings, laboratory results, imaging reports, urological opinion, and treatment response. The urological causes were classified into different categories such as ureteric calculus with ureteric colic, renal calculus presenting with acute pain, acute pyelonephritis, obstructive uropathy, acute urinary retention, pyonephrosis, renal or ureteric trauma, and testicular torsion presenting as lower abdominal pain. Other less common urological conditions that presented as acute abdomen were also recorded wherever identified.

Treatment and management

The management of each patient was planned according to the final diagnosis, severity of symptoms, presence of infection, degree of obstruction, renal function status, and overall clinical condition. Patients with uncomplicated ureteric colic or mild urinary tract infection were managed conservatively with analgesics, hydration, antibiotics where indicated, antiemetics, alpha-blockers, and observation.

Patients with urinary retention underwent urethral catheterisation or suprapubic catheterisation depending on the clinical situation. Those with obstructive uropathy, infected hydronephrosis, pyonephrosis, or deteriorating renal function required urgent urological intervention such as ureteric stenting or percutaneous nephrostomy. Patients with stone disease requiring definitive treatment were managed with endoscopic or surgical procedures as appropriate. Cases of testicular torsion were treated as surgical emergencies, and scrotal exploration with orchidopexy or orchidectomy was performed depending on testicular viability.

Outcome measures

The main outcomes assessed in this study included the spectrum of urological conditions presenting as acute abdomen, the frequency of cases initially suspected as general surgical emergencies, and the common clinical features that created diagnostic confusion. The study also assessed the usefulness of urine examination, renal function tests, ultrasonography, and computed tomography in establishing the correct diagnosis.

Additional outcome measures included the number of patients managed conservatively, the number requiring urological intervention, the need for emergency decompression or surgery, and the clinical condition at the time of discharge. These outcomes helped in understanding the practical diagnostic and management challenges encountered in general surgical emergency practice.

Statistical analysis

Data were first entered in Microsoft Excel (Microsoft Corp., Redmond, WA, USA) and reviewed for completeness and consistency. The cleaned data were then analysed using IBM SPSS Statistics for Windows, Version 28 (Released 2021; IBM Corp., Armonk, New York, United States). Descriptive statistics were used to present the main findings. Categorical variables, including sex, presenting symptoms, provisional diagnosis, final urological diagnosis, imaging findings, treatment approach, and clinical outcome, were reported as frequencies and percentages.

Continuous variables, such as age and duration of symptoms, were expressed as mean with standard deviation when normally distributed. For variables with skewed distribution, median with range was used. Where relevant, associations between clinical features, diagnostic methods, treatment pattern, and final diagnosis were assessed using suitable statistical tests. The results were presented in tables, figures, and descriptive summaries. A p-value <0.05 was considered statistically significant.

Ethical considerations

The study was conducted after obtaining approval from the Institutional Ethics Committee of Chalmeda AnandRao Institute of Medical Sciences (CAIMS/IEC/DRAFT/2024/098). Written informed consent was obtained from all participants before enrolment. In case of minors or patients unable to provide consent, consent was obtained from parents, guardians, or legally acceptable representatives.

Confidentiality of patient information was strictly maintained throughout the study. Personal identifiers were not disclosed in data analysis or reporting. The study did not interfere with routine emergency care, and all patients received standard clinical management according to their diagnosis and condition. The collected data were used only for academic and research purposes.

## Results

Demographic profile of the study population

A total of 80 patients who presented to the general surgical emergency department with features of acute abdomen and were later confirmed to have urological causes were included in the study. The age of the patients ranged from 12 to 78 years, with a mean age of 41.6 ± 15.2 years. The highest number of cases was observed in the 31-50 years age group, which included 34 patients (42.5%), followed by the 51-70 years age group with 21 patients (26.3%).

Male predominance was noted in the study population. Among the 80 patients, 56 (70%) were male subjects and 24 (30%) were female subjects, with a male-to-female ratio of 2.3:1. This pattern may be related to the higher occurrence of ureteric calculus, urinary retention, urological trauma, and testicular torsion among male patients. The age-wise distribution between the genders was not statistically significant, as shown in Table 1.

**Table 1:** Age and sex distribution of the study population Chi-square value: 0.27; p-value: 0.965. No statistically significant association was observed between age group and sex distribution.

Age group	Male, n (%)	Female, n (%)	Total, n (%)
≤20 years	7 (8.8)	3 (3.8)	10 (12.5)
21-30 years	10 (12.5)	5 (6.3)	15 (18.8)
31-50 years	23 (28.8)	11 (13.8)	34 (42.5)
51-70 years	14 (17.5)	7 (8.8)	21 (26.3)
Total	56 (70.0)	24 (30.0)	80 (100.0)

Presenting clinical features

Acute abdominal pain was the universal presenting complaint and was observed in all 80 patients (100%). However, the site, character, and radiation of pain varied according to the underlying urological condition. Flank pain was reported in 36 patients (45%), mainly among those with ureteric calculus and obstructive uropathy. Vomiting was present in 34 patients (42.5%), while fever was documented in 26 patients (32.5%), particularly in patients with acute pyelonephritis and infected obstruction.

Classical urinary symptoms were not present in all cases. Dysuria was observed in 23 patients (28.7%), and hematuria was noted in 17 patients (21.2%). This finding is clinically important because the absence of obvious urinary symptoms may lead to an initial suspicion of a gastrointestinal or general surgical emergency. The distribution of presenting symptoms is shown in Table 2.

**Table 2:** Distribution of presenting symptoms Many patients had more than one symptom at presentation. Therefore, the total percentage is more than 100.

Presenting symptom	Number of patients	Percentage
Abdominal pain	80	100.0
Flank pain	36	45.0
Vomiting	34	42.5
Fever	26	32.5
Dysuria	23	28.7
Hematuria	17	21.2
Abdominal distension	12	15.0
Urinary retention	8	10.0
Groin or scrotal pain	7	8.8

**Figure 1:**
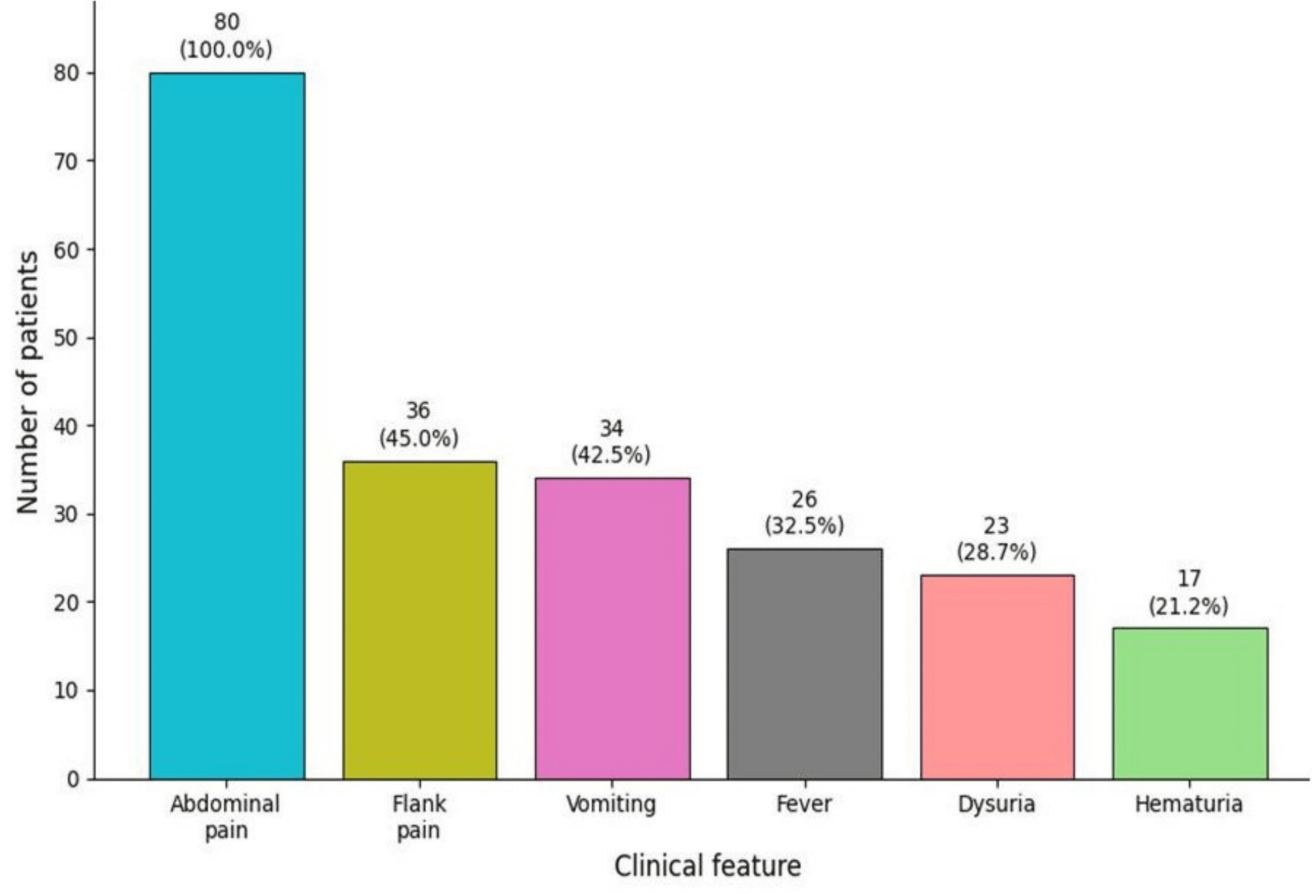
Presenting clinical features among the patients (n=80)

Figure 1 shows that although abdominal pain was present in all patients, urinary complaints such as dysuria and hematuria were seen in a smaller proportion, highlighting the diagnostic difficulty in emergency assessment.

Spectrum of final urological diagnosis

The most common urological condition mimicking acute abdomen was ureteric calculus with ureteric colic, which was diagnosed in 36 patients (45.0%). The next common diagnosis was acute pyelonephritis, seen in 14 patients (17.5%), followed by obstructive uropathy in 11 patients (13.8%). Acute urinary retention was observed in eight patients (10.0%), renal or ureteric trauma in six patients (7.5%), and testicular torsion presenting as lower abdominal pain in five patients (6.2%).

Ureteric calculus was the leading diagnosis in both sexes, but some conditions were seen only among males, including acute urinary retention and testicular torsion. The sex-wise distribution of final urological diagnoses is presented in Table 3.

**Table 3:** Final urological diagnosis according to the patient's sex Chi-square value: 8.50; p-value: 0.131. Percentages were calculated from the total sample size. Testicular torsion and acute urinary retention were observed only among male patients. The difference in diagnostic distribution between males and females was not statistically significant.

Final diagnosis	Male, n (%)	Female, n (%)	Total, n (%)
Ureteric calculus with ureteric colic	24 (30.0)	12 (15.0)	36 (45.0)
Acute pyelonephritis	7 (8.8)	7 (8.8)	14 (17.5)
Obstructive uropathy	8 (10.0)	3 (3.8)	11 (13.8)
Acute urinary retention	8 (10.0)	0 (0.0)	8 (10.0)
Renal or ureteric trauma	4 (5.0)	2 (2.5)	6 (7.5)
Testicular torsion	5 (6.2)	0 (0.0)	5 (6.2)
Total	56 (70.0)	24 (30.0)	80 (100.0)

**Figure 2:**
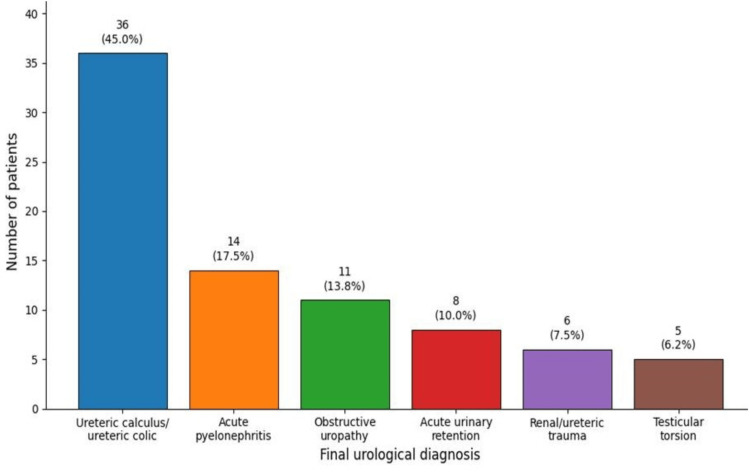
Distribution of urological causes mimicking acute abdomen

The overall distribution of urological causes is also illustrated in Figure 2. 

Initial clinical impression and diagnostic difficulty

At the time of first assessment in the general surgical emergency unit, 31 patients (38.8%) were initially suspected to have a primary general surgical condition rather than a urological disorder. The common provisional diagnoses included appendicitis-like presentation, nonspecific acute abdomen, intestinal colic, biliary colic, and suspected intestinal obstruction.

The diagnostic difficulty was greater in patients without typical urinary complaints. Ureteric colic sometimes resembled appendicitis or intestinal colic, particularly when pain was localized to the lower abdomen. Acute pyelonephritis presenting with fever, abdominal tenderness, and leukocytosis was occasionally confused with intra-abdominal infection. Testicular torsion in young males initially presented with lower abdominal pain and was diagnosed only after focused genital examination and Doppler evaluation. The relationship between initial clinical impression and final diagnosis is shown in Table 4.

**Table 4:** Initial clinical impression in relation to final diagnosis Chi-square value: 6.34; p-value: 0.275. Row percentages were calculated according to each final diagnosis. Although several patients were initially suspected to have non-urological acute abdomen, the association between initial impression and final diagnosis was not statistically significant.

Final diagnosis	Initially suspected surgical abdomen, n (%)	Initially suspected urological cause, n (%)	Total
Ureteric calculus with ureteric colic	10 (27.8)	26 (72.2)	36
Acute pyelonephritis	7 (50.0)	7 (50.0)	14
Obstructive uropathy	5 (45.5)	6 (54.5)	11
Acute urinary retention	2 (25.0)	6 (75.0)	8
Renal or ureteric trauma	4 (66.7)	2 (33.3)	6
Testicular torsion	3 (60.0)	2 (40.0)	5
Total	31 (38.8)	49 (61.2)	80

Laboratory findings

Laboratory investigations provided important supportive evidence in many patients. A raised total leukocyte count was seen in 29 patients (36.3%), mainly among those with acute pyelonephritis, infected obstruction, trauma, and systemic inflammatory response. Microscopic hematuria was detected in 24 patients (30.0%), particularly in ureteric calculus and trauma-related cases. Pyuria was present in 21 patients (26.3%), mostly in patients with urinary tract infection, acute pyelonephritis, and infected obstruction.

Renal function impairment, reflected by raised serum creatinine, was observed in 15 patients (18.8%). This was more common in patients with obstructive uropathy, urinary retention, and infection associated with obstruction. A positive urine culture was found in 13 patients (16.3%), while electrolyte imbalance was noted in nine patients (11.3%). The laboratory abnormalities are summarized in Table 5.

**Table 5:** Important laboratory abnormalities Laboratory findings were interpreted along with clinical examination and imaging. A normal urine report did not exclude a urological cause, especially in early ureteric obstruction or atypical presentations.

Laboratory abnormality	Number of patients	Percentage
Raised total leukocyte count	29	36.3
Microscopic hematuria	24	30.0
Pyuria	21	26.3
Raised serum creatinine	15	18.8
Positive urine culture	13	16.3
Electrolyte imbalance	9	11.3

Imaging findings and diagnostic contribution

Ultrasonography of the abdomen and pelvis was performed as the first-line imaging investigation in all patients. It contributed to the diagnosis in 58 patients (72.5%). The common ultrasound findings included hydronephrosis, renal calculi, ureteric dilatation, bladder distension, urinary retention, and features suggestive of pyelonephritis or pyonephrosis.

CT was used selectively when ultrasound findings were unclear or when the clinical picture remained confusing. Non-contrast CT kidney, ureter, bladder (KUB) was performed in 24 patients and was diagnostically useful in 22 patients (91.7%), especially for localising ureteric calculi and identifying the level of obstruction. Contrast-enhanced CT abdomen was performed in five patients, particularly in trauma or complicated cases, and contributed to diagnosis in all cases. Scrotal Doppler was performed in five clinically suspected cases and confirmed testicular torsion in all. The diagnostic role of different imaging modalities is shown in Table 6.

**Table 6:** Role of imaging in diagnosis Chi-square value: 14.82; p-value: 0.005. Diagnostic contribution indicates that the imaging modality directly supported or confirmed the final diagnosis. CT kidney, ureter, bladder (KUB) was mainly used for suspected urinary calculi, while scrotal Doppler was restricted to patients with suspected testicular torsion. The difference in diagnostic contribution across imaging modalities was statistically significant.

Imaging modality	Number evaluated	Diagnostic contribution, n (%)
Ultrasonography abdomen and pelvis	80	58 (72.5)
Non-contrast CT KUB	24	22 (91.7)
Contrast-enhanced CT abdomen	5	5 (100.0)
X-ray abdomen/chest	18	6 (33.3)
Scrotal Doppler	5	5 (100.0)

Treatment pattern

Out of 80 patients, 44 patients (55%) improved with conservative treatment, while 36 patients (45%) required urological intervention. Conservative management included analgesics, hydration, antibiotics, antiemetics, alpha-blockers, bladder care, and observation. Most uncomplicated ureteric colic cases and mild acute pyelonephritis cases were managed without invasive intervention.

Urological intervention was required mainly in patients with obstructive uropathy, acute urinary retention, infected obstruction, and testicular torsion. The procedures included urethral catheterisation, suprapubic catheterisation, ureteric stenting, percutaneous nephrostomy, endoscopic stone management, and scrotal exploration. Treatment pattern showed a statistically significant association with final diagnosis, as shown in Table 7.

**Table 7:** Treatment pattern according to final diagnosis

Final diagnosis	Conservative management, n (%)	Urological intervention, n (%)	Total
Ureteric calculus with ureteric colic	25 (69.4)	11 (30.6)	36
Acute pyelonephritis	13 (92.9)	1 (7.1)	14
Obstructive uropathy	1 (9.1)	10 (90.9)	11
Acute urinary retention	0 (0.0)	8 (100.0)	8
Renal or ureteric trauma	5 (83.3)	1 (16.7)	6
Testicular torsion	0 (0.0)	5 (100.0)	5
Total	44 (55.0)	36 (45.0)	80

**Figure 3:**
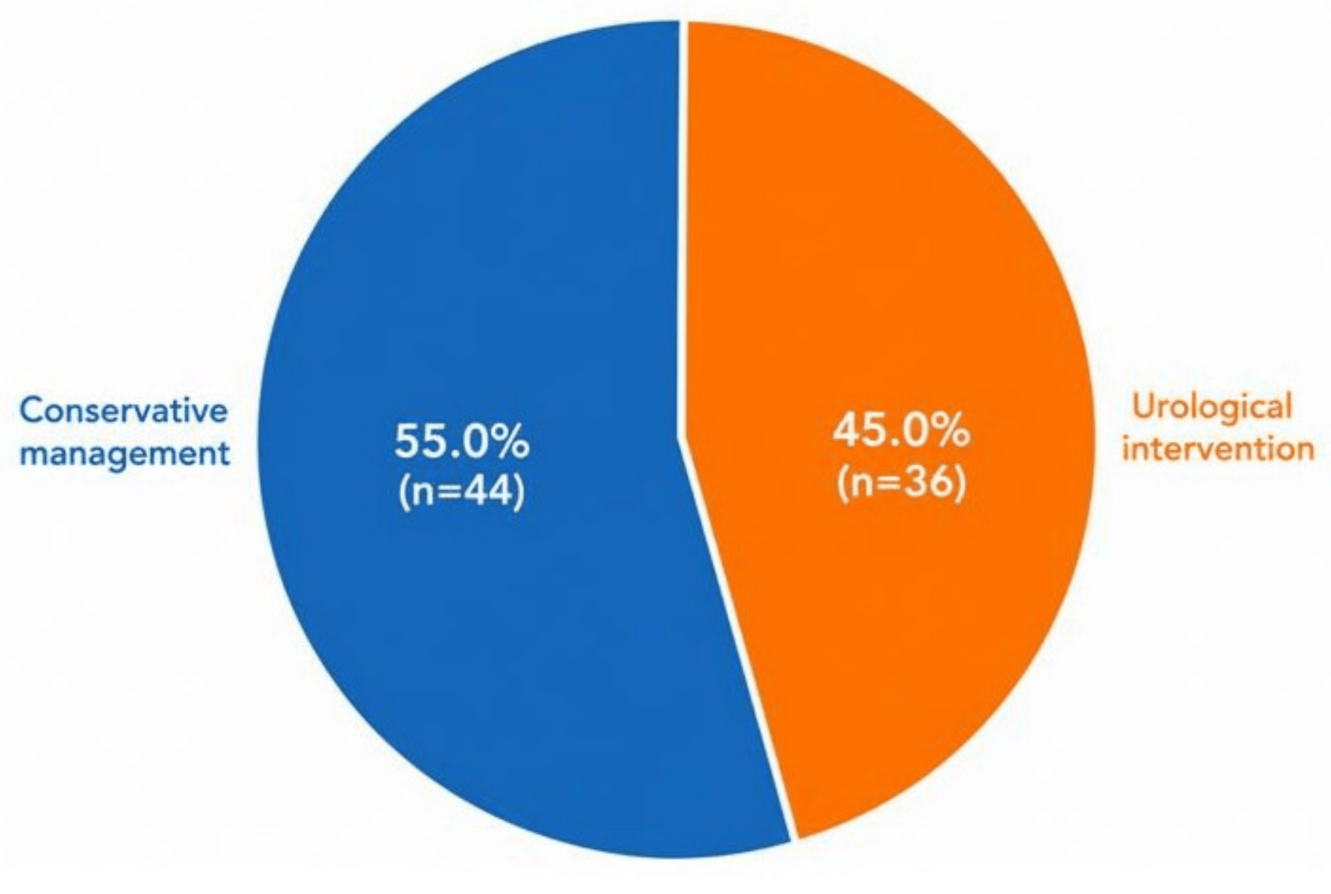
Treatment pattern among patients with urological conditions mimicking acute abdomen

The overall treatment distribution is presented in Figure 3.

This figure shows that slightly more than half of the patients were managed conservatively, while a substantial proportion required active urological intervention.

Clinical outcome

Most patients had favorable clinical outcomes after appropriate diagnosis and management. Seventy-four patients (92.5%) improved and were discharged in stable condition. Four patients (5%) required prolonged hospital stay because of infection, renal dysfunction, or trauma-related complications. Two patients (2.5%) were referred for higher urological care. Of these two referred patients, one had complex renal/ureteric trauma requiring advanced reconstructive urological management, and the other was an adolescent with testicular torsion who required specialized pediatric urological care. These facilities were not available round-the-clock at our institution at the time of presentation. Both patients were clinically stabilised before referral and transferred to higher centres for definitive management. No mortality was recorded in the study population (Table 8). 

**Table 8:** Clinical outcome of the patients (n=80) Outcome was assessed at the time of discharge or referral. Patients referred to higher centres were stable at the time of referral.

Outcome	Number of patients	Percentage
Improved and discharged	74	92.5
Prolonged hospital stay	4	5.0
Referred for higher urological care	2	2.5
Mortality	0	0.0

## Discussion

The present study assessed urological conditions presenting as acute abdomen in general surgical emergency practice. The findings show that urological disorders may closely resemble common surgical emergencies, particularly when urinary symptoms are absent or less obvious. Among 80 patients, ureteric calculus with ureteric colic was the most common diagnosis, followed by acute pyelonephritis, obstructive uropathy, acute urinary retention, renal or ureteric trauma, and testicular torsion. Nearly two-fifths of the patients were initially suspected to have a primary general surgical condition, reflecting the diagnostic challenge in emergency settings.

Acute abdomen is commonly evaluated with suspicion of appendicitis, intestinal obstruction, perforation, pancreatitis, or biliary disease. However, urinary tract diseases are also important causes of acute abdominal pain and should be considered during initial assessment [1-4]. Pain from the kidney, ureter, bladder, prostate, or testis may be referred to the abdomen because of shared visceral innervation. This explains why ureteric colic, pyelonephritis, urinary retention, and testicular torsion may present with abdominal pain, vomiting, fever, guarding, or lower abdominal tenderness [3,4].

Most patients in this study were males, and the commonest age group was 31-50 years. This pattern may be related to the higher occurrence of stone disease, urinary retention, trauma, and testicular torsion among male patients. Similar patterns have been reported in studies on urological emergencies, where renal colic, obstructive uropathy, retention, and genital emergencies are frequently seen in males [7,8]. However, female patients also presented with stone disease, pyelonephritis, and obstruction, showing that urological causes should not be overlooked in either sex.

Ureteric calculus was the leading diagnosis, accounting for 45% of cases. The site of pain in ureteric obstruction depends on the level of the stone. Lower ureteric calculi may produce right or left iliac fossa pain and may be mistaken for appendicitis, intestinal colic, pelvic pathology, or diverticular disease. Although hematuria, flank pain, and radiation to the groin are helpful clues, these findings may not be present in all cases. Therefore, absence of hematuria should not exclude ureteric calculus when the clinical suspicion is strong [5,6].

Acute pyelonephritis and obstructive uropathy were also important causes in this study. Patients with pyelonephritis may present with fever, abdominal pain, vomiting, leukocytosis, and pyuria, which may resemble intra-abdominal infection. Obstruction with infection is particularly serious because it can rapidly progress to sepsis and renal dysfunction. Current recommendations support early imaging and urgent decompression by ureteric stenting or percutaneous nephrostomy when infected obstruction is suspected [5,10].

Acute urinary retention presented with lower abdominal pain and suprapubic distension. In elderly male patients, retention is often related to prostatic enlargement, but urethral stricture, neurogenic bladder, clot retention, drugs, and malignancy should also be considered. A simple suprapubic examination and catheterisation can quickly establish the diagnosis and relieve symptoms [11]. In the present study, all cases of acute urinary retention required urological intervention.

Testicular torsion was another important mimic, particularly among young males. It may initially present as lower abdominal pain, groin pain, nausea, or vomiting rather than obvious scrotal pain. Delay in genital examination may result in missed diagnosis and reduced testicular salvage. Previous reports have also emphasised that testicular torsion should be considered in young males with unexplained lower abdominal pain [12,13].

Laboratory investigations were useful but not diagnostic in isolation. Raised leukocyte count, pyuria, microscopic hematuria, positive urine culture, and raised serum creatinine provided supportive evidence. Hematuria was helpful in stone disease and trauma, while pyuria and culture positivity supported infection. Raised creatinine suggested obstruction, retention, dehydration, or sepsis. These parameters were most useful when interpreted along with clinical findings and imaging.

Imaging played a major role in confirming the diagnosis. Ultrasonography was useful as the first-line investigation because it detected hydronephrosis, renal calculi, bladder distension, urinary retention, and pyonephrosis in many cases. However, ultrasound may miss small ureteric stones or deep pelvic ureteric calculi. CT was therefore useful in selected patients with inconclusive ultrasound findings, suspected ureteric calculus, trauma, or unclear abdominal pathology. Previous studies have shown that CT improves diagnostic accuracy in renal colic and acute abdominal pain, although radiation exposure should be considered carefully [14-17].

A key finding of the study was that 38.8% of patients were initially suspected to have a primary surgical abdomen. This highlights the real-world difficulty of differentiating urological mimics from gastrointestinal emergencies at first contact. Similar observations have been reported in studies of acute abdominal pain, where extra-abdominal and urinary causes were found among patients initially evaluated for surgical abdomen [1-4].

Regarding management, 55% of patients improved with conservative treatment, while 45% required urological intervention. Conservative care was mainly sufficient for uncomplicated ureteric colic and mild pyelonephritis. Interventions were more commonly required in obstructive uropathy, urinary retention, infected obstruction, and testicular torsion. The statistically significant association between final diagnosis and treatment requirement indicates that early and accurate diagnosis has a direct impact on management.

Most patients had a favorable outcome, with 92.5% discharged in stable condition. A small number required prolonged hospital stay or referral for advanced urological care. No mortality was recorded. Timely clinical assessment, appropriate imaging, and early urological referral may have contributed to these favourable outcomes. Further multicentric studies with a larger patient population are recommended to validate these findings and to better understand the pattern of urological conditions mimicking acute abdomen across different hospital settings.

Limitations

The present study has some limitations. It was carried out at a single institution with a relatively limited sample size, so the findings may mainly represent the patient profile, emergency workflow, and referral pattern of this hospital. Diagnostic decisions in an emergency setting may also depend on the availability of imaging facilities, access to timely urological opinion, and the clinical experience of the attending physician. In some patients, ultrasonography was the only imaging modality used, while CT was reserved for selected cases. This may have affected the level of diagnostic confirmation in a few presentations. The study also did not include long-term follow-up; therefore, recurrence of stone disease, delayed complications, and later renal outcomes could not be assessed. Even with these limitations, the study provides useful practical information on urological conditions that can present like acute abdomen in general surgical emergency practice.

## Conclusions

The present study highlights that urological conditions form an important group of disorders that can closely mimic acute abdomen in general surgical emergency practice. Ureteric calculus with ureteric colic was the most common cause, followed by acute pyelonephritis, obstructive uropathy, acute urinary retention, renal or ureteric trauma, and testicular torsion. A considerable proportion of patients were initially suspected to have primary surgical abdominal disease, mainly because classic urinary symptoms were absent or less prominent in many cases. Careful clinical assessment, focused urinary history, renal angle and suprapubic examination, genital examination in young males, urine analysis, renal function testing, ultrasonography, and selective use of CT can improve diagnostic accuracy. Early recognition of these urological mimics helps in timely referral, appropriate intervention, avoidance of unnecessary surgical procedures, and better patient outcomes.
